# Verifying Elimination Programs with a Special Emphasis on Cysticercosis Endpoints and Postelimination Surveillance

**DOI:** 10.1155/2012/974950

**Published:** 2012-11-14

**Authors:** Sukwan Handali, Yudi Pawitan

**Affiliations:** ^1^Division of Parasitic Diseases and Malaria, Center for Global Health, Centers for Disease Control and Prevention, Atlanta, GA 30329, USA; ^2^Department of Medical Epidemiology and Biostatistics, Karolinska Institutet, 17177 Stockholm, Sweden

## Abstract

Methods are needed for determining program endpoints or postprogram surveillance for any elimination program. Cysticercosis has the necessary effective strategies and diagnostic tools for establishing an elimination program; however, tools to verify program endpoints have not been determined. Using a statistical approach, the present study proposed that taeniasis and porcine cysticercosis antibody assays could be used to determine with a high statistical confidence whether an area is free of disease. Confidence would be improved by using secondary tests such as the taeniasis coproantigen assay and necropsy of the sentinel pigs.

## 1. Introduction

Neglected tropical diseases (NTDs) are the most common infections of the world's poorest people and the leading causes of chronic disability and poverty in low- and middle-income countries [1, 2]. As a result, global health policy makers have identified NTD control as a key element central to any strategy designed to achieve the United Nations millennium development goals (MDGs) for sustainable poverty reduction [2]. Several large-scale interventions to control and then to eliminate lymphatic filariasis, leprosy, onchocerciasis, schistosomiasis, helminthiasis, trachoma, and yaws have been conducted [1]. 

 Molyneux et al. [3] defined control as the reduction of disease incidence, elimination as the reduction to zero incidence of a specified disease in a defined geographical area, and eradication as the permanent reduction to zero of the worldwide incidence of infection. However, the methods to measure disease reduction, elimination, or eradication are not established for many NTDs [1]. For lymphatic filariasis and schistosomiasis campaigns, antibody detection has been proposed as a measure of program success [3–6].

**Figure 1:**
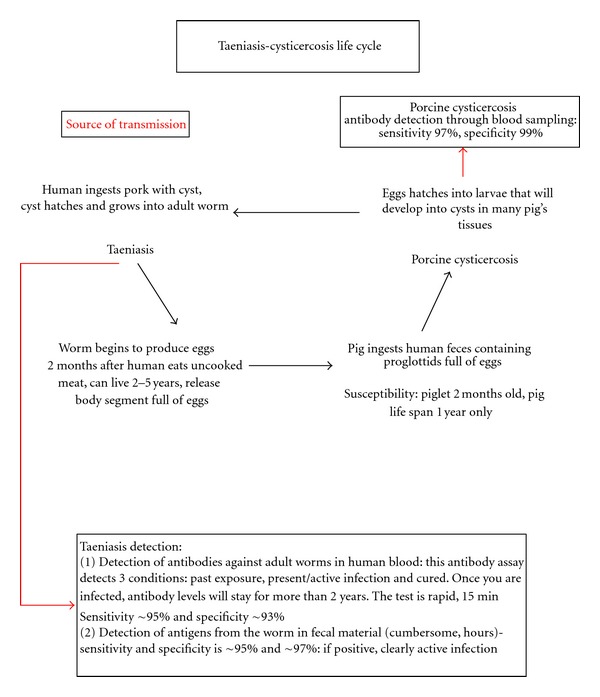
Taeniasis-cysticercosis life cycle and means of verification.

 Cysticercosis, caused by *Taenia solium*, is one of the parasitic diseases that has been deemed eradicable [6]. Strategies for the elimination of cysticercosis have been tested extensively in Peru. Some of the variables evaluated included mass treatment of taeniasis cases, treatment of pigs, and pig vaccination [7–13]. Successful elimination of cysticercosis will probably require that two conditions reach the zero level: the prevalence of human taeniasis and porcine cysticercosis (Figure 1). Once the interventions are deemed effective, the next step is the selection of methods to measure program success. An extensive array of laboratory tests exists for the detection of human and porcine cysticercosis and taeniasis. The value of any of these tools for program verification is unknown. Furthermore, the occurrence of false positives and false negatives from these assays complicates the decision process.

Methods that detect stage-specific antibodies for cysticercosis and taeniasis are available and perform well, yet it is important to know if these serological diagnostic tests are sufficient methods for verifying the elimination of cysticercosis. The available serological test for taeniasis detects antibodies against the *T. solium *adult worm antigen. The most sensitive diagnostic method for porcine cysticercosis is antibody detection using the lentil lectin glycoprotein (LLGP) based enzyme immunoelectrotransfer blot (EITB); however, passive transfer of maternal antibody is a confounder when testing native pig populations in endemic areas [14–17]. To overcome the dilemma posed by maternal antibody, antibody testing in sentinel pigs can be used to determine the incidence of porcine cysticercosis [13]. Although other laboratory tests for cysticercosis and taeniasis are available that could be used to measure program success, the fewer the tests the better. 

Filariasis control programs [18] have suggested that statistical criterion for targets should be defined and that sample sizes should be calculated to provide 95% confidence that the true rate is less than a small target value. While the ideal is a 100% guarantee that the prevalence target is zero, in practice such guarantee is not achievable as that would require testing all individuals with a perfect assay, that is, an assay with no false positives or negatives. The target value should be below the rates that are needed for sustained transmission of lymphatic filariasis [18]. This idea is actually the basis for the Lot Quality Assurance testing that has been used for onchocerciasis elimination programs [19] and schistosomiasis control programs [20]. To provide some guidelines on how elimination programs of taeniasis and porcine cysticercosis are assessed, it is important to calculate the statistical limit and the sample size in population surveys of these diseases.

## 2. Material and Methods

### 2.1. Sensitivity and Specificity

The sensitivity of a test is defined as the ability of the test to correctly identify those with disease and specificity defined as the ability to correctly identify those with no disease. Both are determined by well-defined specimens, truly positive (disease-positive based on the reference or gold standard diagnosis) and truly negative specimens. For taeniasis, the reference standard for diagnosis is identification of *T. solium* eggs or proglottids in stool specimens and/or recovery of partial or intact worms after administration of a purgative. For porcine cysticercosis, the reference standard diagnosis is reached when cysticerci are found in thoroughly-examined pig carcasses by using necropsy. Truly negative specimens are usually obtained from healthy subjects from nonendemic countries and uninfected controls and from subjects with heterologous infections, such as other helminthic infections.

 Based on evaluations using sera that meet these reference standard definitions of true positives and negatives [21, 22], the taeniasis assay has a sensitivity of 94.5% and specificity of 96%. The assay for porcine cysticercosis has a sensitivity of 94% and a specificity of 98.5%.

### 2.2. Upper Limit of Positive Samples Allowed

Based on the ideas from lymphatic filariasis and onchocerciasis control efforts [18, 19], assay performance indices, and given the number of tested samples, we calculated the maximum number of positive tests allowed such that there would be a high confidence (say 99%) that the prevalence is below the target rate (Table 1). In the calculation below we assume that we have a simple random sample, so the standard binomial model applies.

We used the following notations:

 
*a* = 1-specificity
 
*b* = Sensitivity
 
*c* = Confidence level
 
*N* = Sample size
 
*p* = Unknown prevalence of the disease
 
*t* = Target limit of prevalence
 
*z* = Normal quantile at probability *c*
 
*R* = The number of positive tests
 
*U* = The upper limit of the number of positive tests.

Theoretically, the number of positive tests *R* is binomial with mean

(1) m=expected  true  positives+expected  false  positives =Npb+N(1−p)a =N[a+(b−a)p].

In order to get an explicit formula, we use the Poisson and normal approximations, so that on observing *R*, the 100c% upper confidence bound for *m* is

(2) R+z  sqrt(R),

and the corresponding upper bound for the prevalence *p* must satisfy

(3) R+z  sqrt(R)=N[a+(b−a)p].

If the goal is 100c% confidence that the prevalence is less than a target limit *t* (say 1%), the corresponding upper limit in the number of positive tests *R* can be found by solving the following equation in terms of *U*:

(4) $$\begin{array}{c} U+z\;\text{sqrt}\left(U\right)=N\left[a+\left(b-a\right)t\right], \end{array}$$

which can be turned into a quadratic equation and readily solved. 

Note that what we guarantee (with confidence > 100c%) is that the prevalence < target limit *t* if the number of positive tests is less than the upper limit *U*. Conversely, when prevalence > target, we guarantee the probability is <1 − *c* (hence a small value, e.g., 0.01) of declaring the region is free of disease. However, because of false positives, even when the true prevalence is zero, there is no guarantee that the number of positive tests *R* will be less than the upper limit *U*. Let *P* be the probability that a region is declared free of disease, so *P* = Pr(*R* < *U*). We can control *P* by increasing the sample size. Once *U* is determined, *P* can be calculated given a true prevalence *p*, where *R* is binomial with mean *m* above. At *p* = 0, we can then determine the sample size required to guarantee that this probability is large enough (e.g., 0.80). Thus, the guarantee that *P* < 1 − *c* at *p* = *t* and *P* > 0.95 (say) at *p* = 0 and at an appropriate sample size is equivalent to the usual power-significance level combination for the usual hypothesis testing setup. In our approach here, “power” is computed at *p* = 0 and “significance level” at *p* = *t*.

## 3. Results

Based on a target of 1% prevalence and the test performance characteristics for the porcine antibody detection test, the LLGP EITB, and the taeniasis antibody test, we determined the maximum number of positive tests allowed for a fixed sample size (Table 1). For example, if 500 subjects are tested for taeniasis, the number of positives must be less than 15. Similarly, for porcine cysticercosis testing, the number of positives must be less than 8 positives to be 99% confident that the prevalence is less than 1%. Since the sensitivities of these two tests are assumed the same, the table shows that higher specificity implies lower threshold value. This means that in practice it is important to know the characteristics of the test before we can determine the threshold. If our estimate of specificity is too low, the threshold will be too high, and we might wrongly declare a region free of disease. In this case, some of the true positives are wrongly considered false. On the other hand, if our estimate of specificity is too high, some of the false positives are considered true and we are more likely to declare a region is not disease-free. An Excel worksheet with easily changeable parameter values is available from the authors.

**Figure 2:**
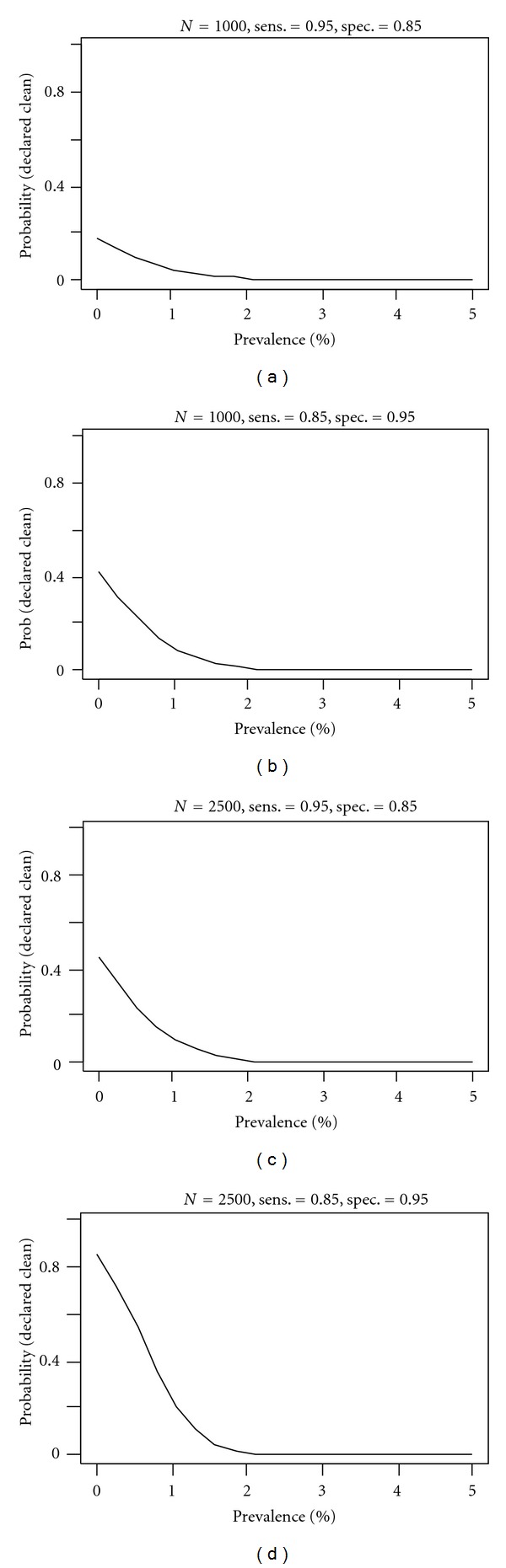
Different sensitivity and specificity scenarios. Each panel shows the probability of declaring a region is free of disease (clean) as a function of true prevalence. It is computed based on 99% confidence at prevalence target 2%. Higher probability at zero prevalence is more desirable.

To assess what sample size is needed in a survey, we can assess the probability of declaring that a region is free of disease given a survey with sample size *N*, where “free of disease” is defined such that the number of positives is less than the upper limit above (see Table 2). Clearly, the probability should be high (close to one) if prevalence is in fact zero. This probability is a function of the underlying prevalence (see Figure 2 for illustration), but by design, the probability is approximately equal to 1 minus the confidence level (=1 − *c*) if prevalence is equal to the target value, and lower if prevalence is larger than the target. For a given sensitivity and specificity of the test, a high probability can be achieved by controlling the sample size. Given a region that is actually disease-free, to get at least 80% probability of declaring it free of taeniasis, the minimum sample size for testing is ~5000, for porcine cysticercosis the minimum is ~2575. 

 Is it better to have a test that is highly sensitive but less specific, or highly specific but less sensitive? We compared the probability of a region declared clean or disease-free at sample sizes of 1000 and 2500, under two different combinations of sensitivity-specificity (Figure 2). It is desirable to have a high value of this probability when the region actually has zero prevalence. The figure shows that there is a greater probability that elimination can be demonstrated using a test with a higher specificity than one with a higher sensitivity. This explains why above we found that taeniasis, whose assay has a lower specificity, requires a larger sample size.

## 4. Discussion

 Although Weill and Ramzy [18] favored antibody assays for the detection of early infection, there are several arguments against using antibody assays for elimination verification. Detection of antibodies can indicate more than one infection state: a current and active infection, a past infection, or simply an exposure. However, assuming high sensitivity, a negative test is informative for no disease transmission. The longevity of antibodies for taeniasis is not known, but it is known that adult *T. solium* worms can live up to two years [23]. Thus, two years after a full elimination of taeniasis, the presence of antibodies should reflect past infections.

 To increase its practical value, it is worth extending our procedure in at least two directions: (i) allow for a cluster sampling procedure and (ii) account for a finite-sample correction. The first extension overcomes the limitations of the simple random sampling design used in our calculations. 

 If necessary to decrease the sample size, a group of sequential testing methods could be used; with this method, if the data show that the probability of being disease-free is either highly probable or highly improbable, then the data collection can be stopped earlier than planned. An advantage in eliminating cysticercosis is that high-performance coproantigen test is also available. Only active, current infections will produce positive results with the coproantigen test. Combining rapid diagnostic tests for taeniasis and coproantigen tests for the positive subjects will give a powerful indicator of the potential absence of transmission of disease.

 With these effective strategies, antibody detection for taeniasis in human subjects and antibody detection for porcine cysticercosis, defining endpoints for control is possible. To reduce the number of samples needed to make determinations for control, additional testing using the coproantigen test in antibody positive taeniasis individuals can be performed.

## Figures and Tables

**Table 1:** Upper limit (*U*) of positive tests allowed for 99% confidence that the prevalence is less than the target limit of 1%. Sensitivity of the tests is assumed equal to 94%.

Sample size	100	500	2500	5000	7500
Taeniasis (specificity 96%)	1	15	99	211	325
Cysticercosis (specificity 98%)	0	8	55	120	187

**Table 2:** Probability of declaring a region is disease-free given that the region has true zero prevalence; a high value of the probability is desirable. Sensitivity is assumed equal to 94%.

Sample size	100	500	2500	5000	7500
Taeniasis (specificity 96%)	8.7%	15.1%	48.6%	79.8%	93.2%
Cysticercosis (specificity 98%)	13.3%	33.1%	78.7%	97.8%	99.9%
